# Ecological Adaptation of Diverse Honey Bee (*Apis mellifera*) Populations

**DOI:** 10.1371/journal.pone.0011096

**Published:** 2010-06-15

**Authors:** Robert Parker, Andony P. Melathopoulos, Rick White, Stephen F. Pernal, M. Marta Guarna, Leonard J. Foster

**Affiliations:** 1 Centre for High-Throughput Biology and Department of Biochemistry and Molecular Biology, University of British Columbia, Vancouver, Canada; 2 Agriculture and Agri-Food Canada, Beaverlodge Research Farm, Beaverlodge, Canada; 3 Statistical Consulting and Research Laboratory, Department of Statistics, University of British Columbia, Vancouver, Canada; University of Sydney, Australia

## Abstract

**Background:**

Honey bees are complex eusocial insects that provide a critical contribution to human agricultural food production. Their natural migration has selected for traits that increase fitness within geographical areas, but in parallel their domestication has selected for traits that enhance productivity and survival under local conditions. Elucidating the biochemical mechanisms of these local adaptive processes is a key goal of evolutionary biology. Proteomics provides tools unique among the major ‘omics disciplines for identifying the mechanisms employed by an organism in adapting to environmental challenges.

**Results:**

Through proteome profiling of adult honey bee midgut from geographically dispersed, domesticated populations combined with multiple parallel statistical treatments, the data presented here suggest some of the major cellular processes involved in adapting to different climates. These findings provide insight into the molecular underpinnings that may confer an advantage to honey bee populations. Significantly, the major energy-producing pathways of the mitochondria, the organelle most closely involved in heat production, were consistently higher in bees that had adapted to colder climates. In opposition, up-regulation of protein metabolism capacity, from biosynthesis to degradation, had been selected for in bees from warmer climates.

**Conclusions:**

Overall, our results present a proteomic interpretation of expression polymorphisms between honey bee ecotypes and provide insight into molecular aspects of local adaptation or selection with consequences for honey bee management and breeding. The implications of our findings extend beyond apiculture as they underscore the need to consider the interdependence of animal populations and their agro-ecological context.

## Introduction

Human association with the Western honey bee (*Apis mellifera* L.) spans at least 7,000 years [1]. At present, this species is largely domesticated and is not only used to produce hive products, such as honey, wax and royal jelly, but is the primary species used for the pollination of agricultural crops globally [2]. *A. mellifera* initially evolved in Africa and then, in at least two separate events predating the arrival of *Homo sapiens*, migrated north to central Asia and northern Europe [3], diverging into at least two-dozen physiologically, behaviourally and morphologically-distinct sub-species [4]. Domestication, however, has eroded sub-species distinctions through hybridization, particularly in regions such as North America where *A. mellifera* was not native.

It is common practice among North American beekeepers to replace queens every one to two years to maximize productivity [5]. These queens originate from a restricted set of queen breeders situated in regions optimal for queen production and mating. In the United States these regions are located in Hawaii, central California and along a south-eastern band spanning from Florida through to Texas. While a small number of queens in Canada are produced domestically, the majority are imported from central California, Hawaii, New Zealand, Australia or Chile. Since the genotypes of the individual workers in the colony are derived from the mated queen, this practice undermines the stock improvement goals of queen purchasers in two ways. First, purchasers frequently value traits differently than queen breeders [6]. Second, the agro-ecological conditions where queens are selected may not resemble those where the queens are used. Combined, these aspects results in a situation where many beekeepers operate without the full benefits of stock improvement.

Like any livestock, the variation in phenotypes observed among honey bees are a product of artificial and natural selection. The common methodology for estimating variation among populations, however, provides only a limited picture of the adaptive significance of this variation. Such methods rely on quantifying neutral genetic variation among populations by correlating microsatellite markers with quantitative traits found in the populations. Consequently, these techniques provide little insight into the biochemical mechanism(s) at work in adaptation [7]. Mutations that occur in protein coding regions are infrequent but can lead to mechanistic insight: in feral honey bees the identification of locally adapted population clines due to geographic diversity has been shown previously by the polymorphism of alloenzymes [8], [9].

Of the large-scale approaches available to study biological diversity, next-generation sequencing technology allows a deep and high-resolution probing of differences among groups or individuals in a species [10] but is too far removed from the level of proteins to provide much functional insight into the adaptations. Even mRNA expression profiling, either by RNA-Seq [11] or more classical microarrays [12], [13], is not consistently correlated with protein expression [14], [15]. Proteomics [16], in contrast, directly measures biomolecules responsible for responding to a changing environment and so is ultimately the best approach for probing the underlying mechanisms at work in adaptation. Despite this potential power, proteomics has been under-utilized in the study of population biology [17] and has not been previously used to study local adaptation among commercial bee populations.

The primary objective of this study was to determine the diversity of protein expression in commercial honey bee populations, to develop an understanding of the mechanisms used by bee populations to adapt to different agro-ecological conditions and to develop tools for bee breeders. Our approach towards this objective was to test the null hypothesis that no differences in expression exist among the populations, given that queen production is centralized in a few locations. In order to address these goals, we carried out a quantitative analysis of the midgut proteome from adult nurse bees. The adult worker midgut was chosen as it is a key organ in a bee's interaction with its environment: it is the primary site of processing for ingested nutrients and toxins, and as the route of entry for enteric pathogens, the midgut is also involved in individual [18] and colony-level [19] resistance to disease. The honey bee gut was also the organ of choice in a recent gene expression study investigating the cause of Colony Collapse Disorder (CCD) [12]. Interestingly, alongside potential markers associated with the prevalence of CCD, the geographical origin of the colonies was shown to affect gene expression. The bees used in our study were all reared and sampled at the Alberta-based Beaverlodge Research Farm from queens imported from diverse geographical locations, including eastern and central Canada, California, Hawaii, Chile and New Zealand. Our findings unveil major differences in the basic biochemical machinery of these bees, especially proteins involved in metabolism, protein processing and translation. These results have major implications for apiculture as they provide a molecular explanation for the common observation that transplanted bees from different climates cannot always adjust well to a new location [20], [21].

## Results

### Population Proteomics

**Figure 1:**
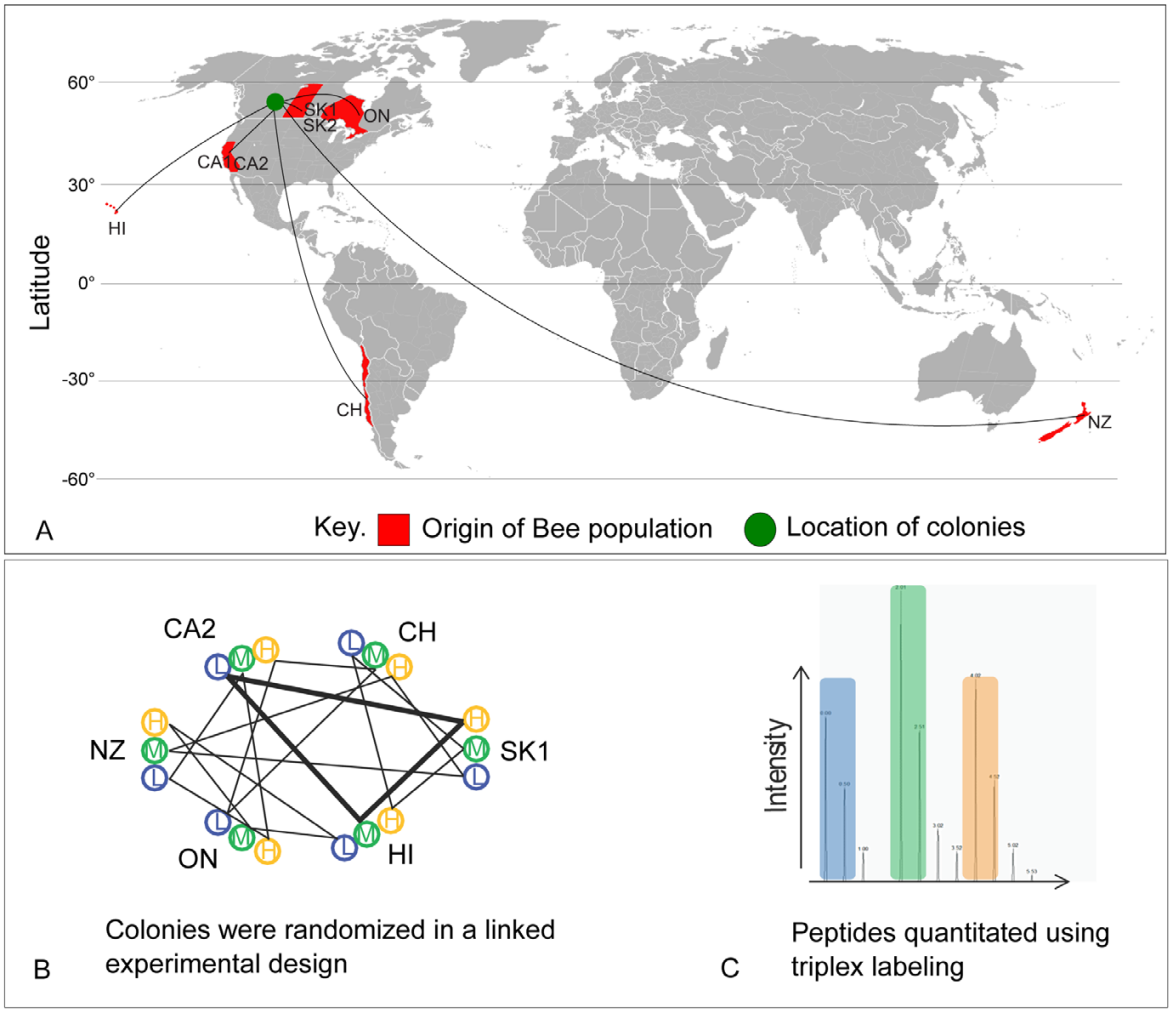
Overview of experimental design. A. World map indicating the approximate geographical location (red land masses) from where bee colonies were originally imported to the sampling site Beaverlodge, AB, Canada (green circle). B. Graphical representation of the incomplete randomized block design (IRBD) used to define the three colonies analyzed in each block (represented by any triangle). The IRBD was restricted so that no two colonies from the same population were in the same block, and so that the differential labels were balanced across colony replicates (the full design implemented can be found in Dataset S1). C. Representative spectra of peptides from triplex labelling experiment. Peptides from the three colonies in each block were differentially labelled with formaldehyde isotopologues (represented here by blue, green and orange, see Methods) before being analyzed by LC-MS/MS. Differential ratios of the labels were then extracted from the spectra, providing a measure of relative abundance for the proteins they originate from (the protein ratios obtained from this workflow for all 58 blocks can be found in Dataset S2).

**Figure 2:**
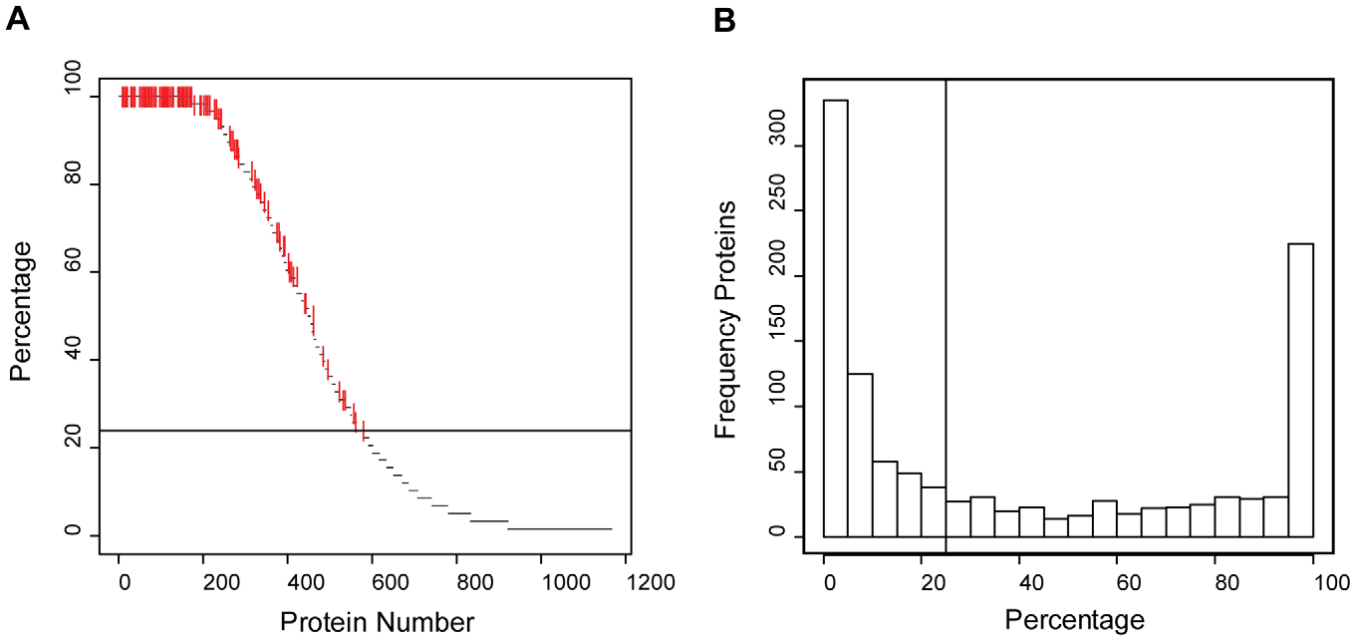
Description of proteomic dataset. A. Frequency (x-axis) at which each protein (y-axis) was observed across all 58 blocks. B. The number of proteins (y-axis) and their observation frequency (x-axis) across all 58 blocks. The horizontal line represents the cut-off applied for keeping proteins in the dataset: only those proteins observed in at least 25% of the blocks were considered further. Of these, 172 proteins (red lines in A) had statistically significant expression patterns with respect to population (*P*<.05).

Table 1 summarizes the eight different sources of honey bees analyzed; each source was given a code that can be used to identify these experimental groups in later figures. A diverse array of bee stocks from locales covering the most popular sources of commercial queens for Canadian beekeepers was investigated (Fig 1a). For the purposes of this study, between four to eleven colonies originating from each location but raised at least one generation in Beaverlodge, AB, were sampled; collectively all the colonies from a single location are referred to as a ‘population’. Sample collection was performed over a two-day span in July 2008, with each colony sampled in triplicate and each replicate comprised of midguts from five nurse bees. We then applied a triplex labeling technology using formaldehyde isotopologues [22] and a randomized incomplete block design (Fig. 1b,c, Data set 1) that would allow us to derive relative expression profiles for all proteins across all colonies. In this approach, a ‘block’ refers to one triplex analysis of bees from three different colonies. Analysis of all 58 blocks by liquid chromatography-coupled tandem mass spectrometry (LC-MS/MS) yielded a rich dataset that was both deep, with more than 470 proteins measured in each block, and broad, with more than 570 proteins detected in at least 14 blocks (Dataset S2). Apart from detecting peptides from proteins several times in different colonies (Dataset S3), confidence in our identification methodology was measured by controlling the false discovery rate (estimated to be about 0.25%) utilizing a decoy strategy. Summary statistics for the entire dataset are displayed in Table 2, Figure 2 and Dataset S3).

**Table 1:** Honey bee populations.

Breeder location	Strain	Population Name	Number of colonies
**Cutknife, SK, Canada**	Unknown	SK1	4
**Cutknife, SK, Canada**	Russian Carniolan	SK2	4
**Apple Hill, ON, Canada**	Russian Carniolan	ON	4
**Hawke's Bay, New Zealand**	Carniolan/Italian	NZ	8
**Captain Cook, HI, USA**	New World Carniolan/Italian	HI	8
**Santiago, Chile**	Unknown	CH	10
**Yuba City, CA, USA**	New World Carniolan	CA1	11
**Orland, CA, USA**	New World Carniolan/Italian	CA2	9

**Table 2:** Summary of Proteomics.

Data feature	Number
Experimental blocks	58
No. Matched spectra ion score = />25	283074
No. Unique Peptides	4735
No. Proteins (1peps)	1454
Quantifiable Proteins (Parsimony rule)	1169
False discovery rate peptide ID (reverse decoy)	0.25%
Quantified Proteins (observed = />25%)	578
Differentially expressed Proteins (p = /<0.05)	172

Using these datasets we then asked, are any of the protein expression profiles explained, at least in part, by an effect of population? As is typical in LC-MS/MS experiments, a significant fraction of the proteins identified were observed in only a small number of blocks so in order to streamline down-stream analyses, we removed from our dataset all those proteins where a quantitative measurement was available in fewer than 25% of all blocks (Fig. 2b). This resulted in a final dataset comprised of 578 proteins, for which a Linear Mixed Effects model was used to estimate the effect of population on the expression level for each protein, adjusting for colony, block and label factors. A statistically significant (*P*<.05) effect of population was found for 172 proteins across the eight populations tested (Fig. 2a, red markers, Dataset S3).

### Gene Ontology assignment and functional enrichment

In order to evaluate the adaptive processes across the populations studied, the 172 population-dependent proteins were categorized according to direction of expression and their enrichment for Gene Ontology (GO) annotations using their FlyBase [23] orthologs. Table 3 and Dataset S4 provide GO (*Drosophila melanogaster* slim) protein annotations under Biological Process, Molecular Function and Cellular Compartment categories for the most significantly enriched annotation observed in each population. Annotations are observed multiple times and show common and bi-directional regulation between populations. Of the processes that are over-represented, protein folding is evident in 4 populations, being up-regulated in both Californian (2^nd^ term found) and down-regulated in all three Canadian populations. Processes for energy production, ion transport and carbohydrate metabolism were also common and bi-directional within populations. Intriguingly, enrichments appeared to counterbalance each other; where processes involving the life span of a protein (e.g. translation, protein folding) are more highly expressed, metabolic processes (e.g. ion transport) are under-expressed, and vice versa. The ontology for cellular component also showed this pattern, where enrichment for mitochondrion is observed always in opposition is cytosol, ER, ribosome or Nucleus.

**Table 3:** Number of differentially expressed proteins and the direction of change in expression for each population.

Popul-ation	Expression	Biological Process	*P* value	Molecular Function	*P* value	Cellular Component	*P* value
**Ch**	↑37	translation	.011	binding	.037	cytosol	.019
	↓45	carbohydrate metabolic process	.0019	catalytic activity	4.2E-05	mitochondrion	.0031
**Ko**	↑12	*No significant enrichment*		nucleic acid binding	.0088	*No significant enrichment*	
	↓33	generation of precursor metabolites and energy	1.3E-05	transporter activity	1.5E-09	mitochondrion	2.7E-05
**NZ**	↑12	*No significant enrichment*		*No significant enrichment*		*No significant enrichment*	
	↓23	*No significant enrichment*		*No significant enrichment*		*No significant enrichment*	
**Ol**	↑42	response to stress	.0029	binding	.025	endoplasmic reticulum	.015
	↓41	ion transport	.00024	transporter activity	6.1E-05	mitochondrion	.00014
**oR**	↑20	electron transport	0.014	catalytic activity	.00022	mitochondrion	1.9E-06
	↓17	protein folding	.0086				
**Pt**	↑30	generation of precursor metabolites and energy	1.2E-08	transporter activity	.0025	mitochondrion	.00010
	↓22	protein folding	.0065	binding	.0061	nucleus	.0073
**sR**	↑25	carbohydrate metabolic process	.016	catalytic activity	.057	mitochondrion	.0026
	↓19	protein folding	.00028	binding	.00036		
**St**	↑36	protein folding	1.2E-05	binding	.056	*No significant enrichment*	
	↓41	ion transport	.0023	transporter activity	.00061	mitochondrion	2.7E-05

GO terms significantly enriched (FDR Corrected *P*>.1 Hypergeometric test).

### Geographical origin regulates gene expression

**Figure 3:**
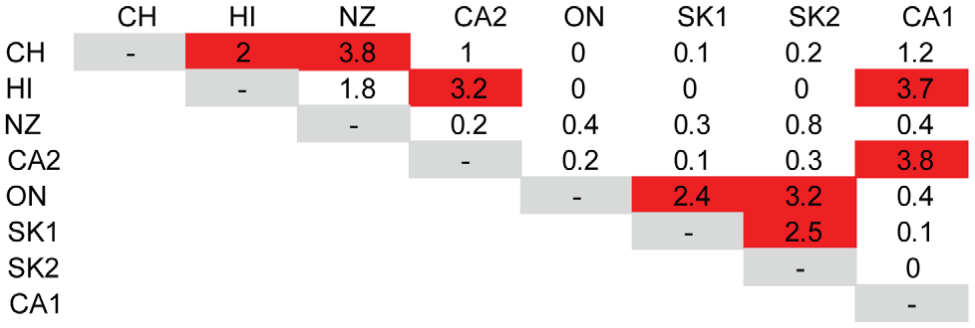
Similarity matrix showing the presence of significant overlaps in protein expression between populations. Data shown is the representation factor where >1 indicates more overlap than expected between two populations. Statistical significance of this overlap was determined by applying an exact hypergeometric test (red indicates *P*<.05).

If the populations studied here had adapted or were optimally bred to survive in the climate in which they were situated prior to transfer to the experimental site then one would expect a higher degree of similarity between colonies from areas with similar climates than between colonies from areas with different climates and, indeed, this was true. Statistically significant (*P*<.05, hypergeometric test for similarity) overlap in protein expression patterns was detected between the New Zealand and Chile populations, between the two Californian populations and between the two Saskatchewan populations (Fig. 3). This finer division of the populations in pairs showed that within each of the similar pairs, two general classes of proteins appeared to dominate: stress response and protein folding chaperones, as well as energy production enzymes, particularly those from the mitochondria. Proteins responsible for protein folding (GO:0006457) were over represented in proteins expressed at higher abundance in the Californian populations, while proteins sharing this GO term as well as stress response components (GO:0006950) were highly enriched among the most highly down-regulated proteins in the Saskatchewan lines. Opposing this, Californian lines tended to have reduced expression of many proteins at the heart of mitochondrial function, including ATP synthase β and δ subunits, cytochrome C oxidases, NADH dehydrogenases and malate dehydrogenase, indicating a much lower rate of primary metabolism in the Californian populations compared to the other populations analyzed. In contrast, enzymes all along Carbohydrate metabolic processes, the citric acid cycle and the oxidative phosphorylation pathway were up-regulated in the Saskatchewan populations, including cytochrome c oxidase and reductase, a citrate synthase, transaldolase and 6-phosphogluconolactonase. Strong overlaps were also observed between both Californian populations versus the Hawaiian population and between all three Canadian populations. Taken together these results indicate that protein expression is regulated by location, but also that parallel regulation may occur in similar climates at diverse locations.

### Metabolic adaptation in geographically distinct bee populations

**Figure 4:**
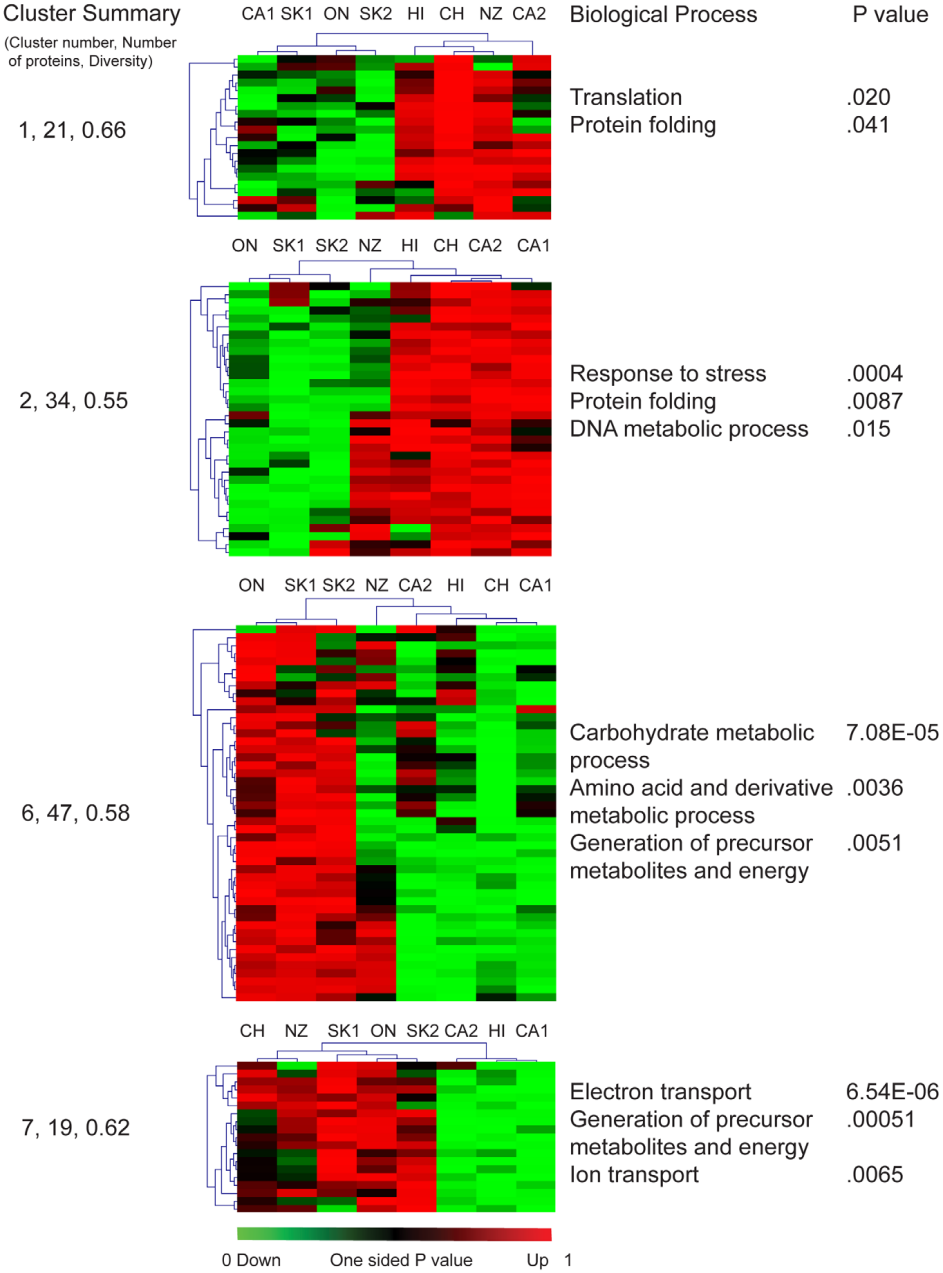
Results of clustering of all midgut proteins found differently expressed for any population. The 172 proteins with a significant population effect were grouped using the SOTA into eight clusters based on their one-sided *P*-values; the four clusters exhibiting significant enrichment in functional classes of proteins are shown. The proteins within each SOTA cluster were further clustered hierarchically by both protein (vertical) and population (horizontal). The cluster number, number of proteins, and diversity is given to the left. GO terms for biological processes found to be significantly enriched (FDR Corrected *P>*.05 Hypergeometric test) in each cluster are shown to the right.

To discover less implicit relationships present between the populations, we used the one-sided *P*-values from the Linear Mixed Effects analysis to carry out inclusive and explorative analysis using a neural network clustering method based on the self-organizing tree algorithm [24]; hierarchical clustering of each cluster then aided the visualization of similarities and differences across all populations. The most efficient analysis was determined empirically and resulted in eight gene clusters of low diversity >0.75. The four largest clusters (<10%) were significantly enriched for proteins with function in specific cellular processes and cellular compartments (Fig. 4). Notably, the largest cluster was highly enriched for basic biochemical pathways, including carbohydrate metabolic process (GO:0005975), amino acid and derivative metabolic process (GO:0006519) and generation of precursor metabolites (GO:0006091); Bi-directional hierarchical clustering divided the populations into two main groups, with the three Canadian populations clustering away from the other five. In concordance with the results from the hypergeometric test for similarity that showed up-regulation of mitochondrial proteins in Canadian populations, the cellular compartment ontology for mitochondrion (GO:0005739) was also significantly enriched in this cluster. Cluster 7 also provided additional confirmation of the mitochondria as the principle site for response to adaptive pressure: this grouping was highly enriched for a separate cluster of mitochondrial proteins, including components of Electron transport (GO:0006118), Generation of precursor metabolites and energy and Ion transport (GO:0006811). Finally, cluster 2 the second largest cluster, terms for Response to stress (GO:0006950), Protein folding and DNA Metabolic processes (GO:0006259) were enriched as was the Cellular component Nucleus with higher expression in Hawaiian, Chilean and Californian populations. Overall the results of this cluster analysis are in agreement with the observations reported above in that the Canadian populations show higher levels of proteins involved in energy metabolism compared with the other populations.

**Figure 5:**
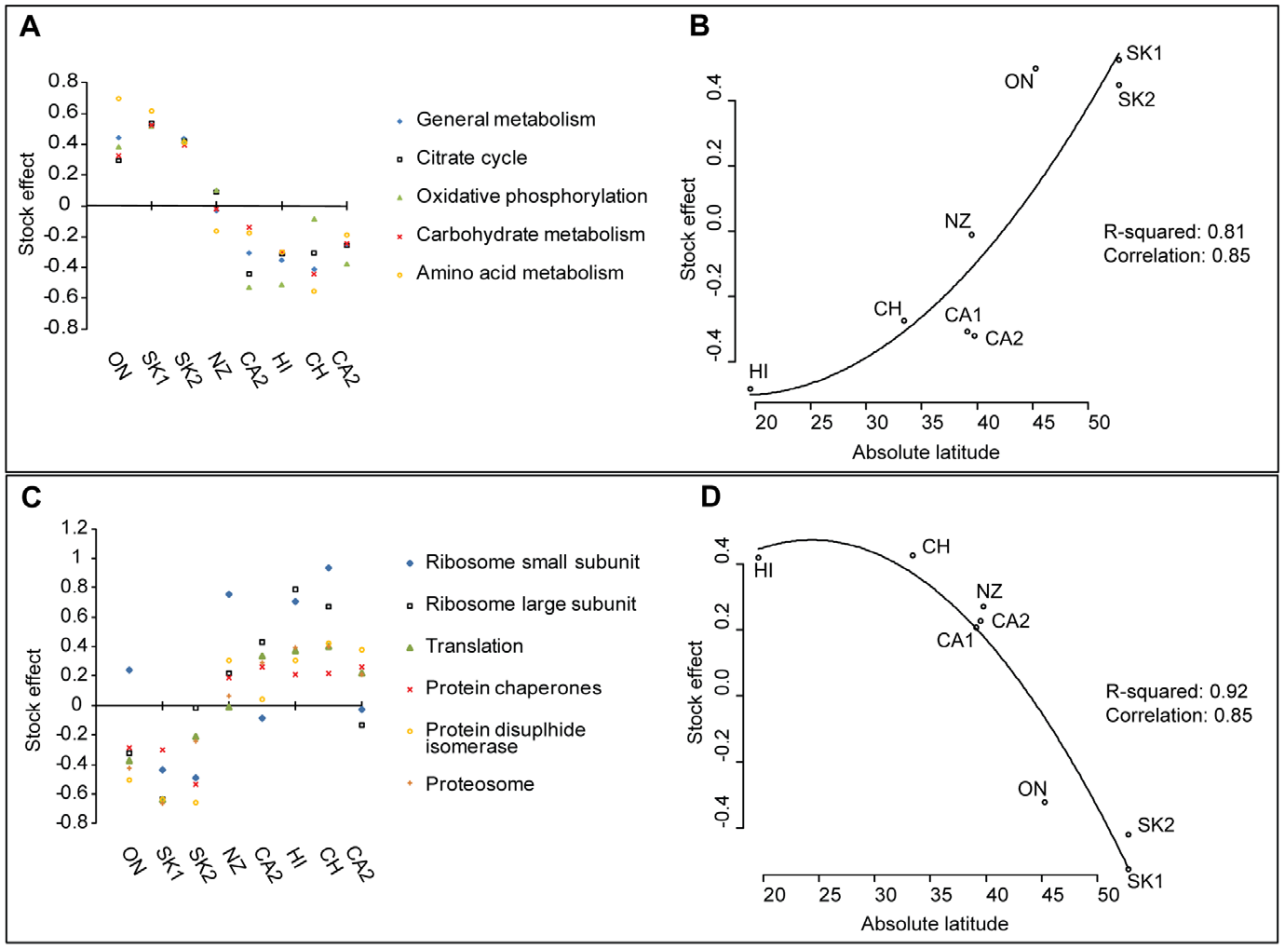
Pathway analysis of honey bee midgut proteins across the populations studied. Bee proteins identified here were first matched against KEGG [25] using the annotations of their *Drosophila* orthologs and then the median protein abundance for each pathway was plotted. A. Proteins were divided into several key metabolic processes. B. Protein biosynthesis/folding/degradation factors exhibited a distinct profile with respect to the populations analyzed. C-D. Linear regression analysis of stock effect versus absolute latitude. The dependant variable (*x*), was median stock effect for all proteins of (C) general metabolism and (D) protein biosynthesis/folding/degradation proteins are plotted against the independent variable (*y*) absolute degrees from equator of population origin. A quadratic model (Y = βo + β1A + β2A2) is fitted to the data and the R^2^ and correlation values shown.

The clustering performed here was based on *P*-values from the Linear Mixed Effects analysis as they consider significance of the relative differences on protein levels. However, a close approximation to protein abundance in the Linear Mixed Effects model is the population effect value, which give a measure of relative change (log scale) of abundance for each protein compared to all the populations (see Methods); the average population effect values for each *Drosophila* KEGG [25] pathway is plotted in Fig. 5a,b. The dme01100 General Metabolism, dmel00480 Citrate cycle and dmel 00190 Oxidative phosphorylation pathways were used, along with two manually constructed composite pathways named Carbohydrate metabolism and Amino acid metabolism. Here functional specificity allows any noise associated with co-clustering proteins of different pathways to be eliminated. From the plot it is clear that populations from Ontario, and Saskatchewan express higher levels of all 4 key metabolic pathways that emerged from the analysis of *P*-value clustering above. Likewise, opposing expression patterns in the population effects are observed for proteins involved in protein biosynthesis/folding/degradation: Ribosome Small subunit, Ribosome large subunit, Translation, Protein Chaperones, Protein disulphide isomerase and Proteosome (Fig. 5b). To confirm the presence of a relationship between local climate and expression of these processes, linear regression was employed to test for a correlation between absolute latitude and protein expression (Fig. 5c,d). The average stock effect for ‘general metabolism’ and all protein biosynthesis/folding/degradation processes are plotted. Regression analysis indicated there was indeed a high correlation between absolute latitude and protein expression for both of these biological processes.

### Potential adaptive mechanisms of gut innate immunity

Among those proteins also enriched in clusters 1 and 2 (Fig. 4) were several proteins involved in gut innate immunity, including Toll-like receptor (TLR) 3, Vanin-1, Ferretin 2 and Chitinase, indicating that bee populations may differ in their ability to mount an immune response or cope with local enteric pathogens. TLR-3 is a pattern recognition receptor involved in the recognition to viral pathogens, and intestinal tissue necrosis during inflammatory responses [26], [27]. Vanin-1 has pantetheinase activity that mediates oxidative burst in the gut epithelia, potentially facilitated via Toll/Cytokine signaling [28], [29]. Ferretin 2 is also involved in the oxidative stress response, in this case it sequesters excess ferric iron, reducing the generation of H_2_O_2_ and abating oxidative stress induced in response to the activity of proteins like Vanin-1 [30]. Chinitases are involved in inflammatory responses of the intestines, inducible by cytokine signalling [31]. Further, insect chitinases modulate the thickness of the peritrophic membrane (PM) which forms a film-like structure separating undigested nutrients from epithelial cells protecting the epithelium from food abrasion and enteric pathogens (reviewed by [32]. The differential expression of proteins involved in the exposure and response to pathogens suggests that different populations may have different levels of susceptibility to disease.

## Discussion

Honey bees are an essential component of human agriculture and many crops are completely dependent on the pollination services provided by bees, often over a period as short as a few days [33]. Superimposed on these demands, is the reality that under northern temperate climatic conditions annual viability of commercial colonies is mitigated without extensive human assistance, e.g., Peace River District of British Columbia and Alberta, Canada, yet may produce large quantities of honey [34]. These demands make for a complex beekeeping paradigm where beekeepers in northern climates are required to purchase queens or bulk bees from breeders who specialize in their production. Because of the high demand for queens in Canada during the spring, many are sourced from offshore sites, particularly from the tropics (e.g., Hawaii) or the southern hemisphere (e.g., New Zealand, Chile). This importation of bees is currently necessary but not optimal as the bees may not perform as well as locally raised bees [21]). Queen breeders endeavour to select and to maintain economically desirable phenotypes (e.g., high honey production, disease resistance, winter survival, gentleness) in their populations, nevertheless when bees are sold abroad the fidelity of these characteristics is not necessarily maintained. Through proteomic analysis of honey bee populations from several geographically distinct regions, our data indicates that optimized metabolic capacities for various climatic regions have developed, potentially conferring beneficial phenotypic characteristics. It is worth noting that the adaptation of the queens to their original breeding site was maintained after being moved to an experimental location in Alberta where their colonies were sampled. These local adaptations observed as differences in protein levels may be related to genetic or epigenetic changes in the queens of the different populations.

Our null hypothesis stated that no differences should exist among the protein expression profiles of different populations of honey bees; the basis for this being that queen production for North America is centralized in a few locations. The data presented here argue strongly in favor of rejecting this null hypothesis: a) At the individual protein level there are at least 172 proteins whose expression in the midgut correlates with population (Fig. 2, Fig. 4). b) At the level of the whole protein expression profile, populations from similar climates tended very strongly to be more similar to one another (Fig. 3). c) At the functional level, the expression levels of whole classes of proteins tended to be co-regulated (Fig. 5, Table 3). We are cognizant, however, that the choice of colonies used in these analyses did not permit random selection from a large cohort representing each population, due to constraints brought upon by practical considerations associated with importation and maintenance of stock. These data nonetheless are highly suggestive of intriguing local adaptations occurring in honey bee metabolism.

The populations studied here may represent separate geographical ecotypes, where metabolic control and protein synthesis/folding mechanisms has been finely tuned to confer fitness to local environmental pressures such as climate, food resources, predation and diseases. In general, such processes are non-random series of genetic events where allelic frequencies alter with a direct influence on the phenotype. It must also be appreciated that these populations were developed by commercial breeding programs and thus environmental factors are not the only parameters affecting phenotype; selective breeding, as well as importation and hybridization of new genetic stock, has likely also influenced each population. Also, selection is not the only mechanism that can result in inter-population variance. Genetic drift is a stochastic mechanism that can change the frequency in alleles within a population regardless of the influence of ecological gradients. Although in this case, the type of micro-evolutionary process responsible is not directly demonstrated, the processes were found to cluster bi-directionally depending on climate of population origin. This indicates an extremely low chance that observations were the result of random genetic drift, as the same protein expression trends appear to be lost or gained in the opposing direction at each geographical origin. Non-random genetic causes, such as direct exchange of genetic material between two or more of the populations studied here can also be ruled out. For example, New Zealand and Chile have national policies in place that restrict the importation of bees yet these two populations showed a high degree of similarity. The cases are less clear-cut between the Californian and Hawaiian populations or between those from Saskatchewan, nevertheless each of these breeders maintains that there has been no intentional genetic exchange among the populations in question. Likewise, even populations who share a traceable common ancestor but who had several years to adapt to their current environment did not show any greater similarity than those sharing a climatic region, e.g., the Ontario and Saskatchewan Russian lines.

In the data presented here, pairs of populations that shared the most similar latitudes tended to have the most similar protein expression profiles. Through the analysis of isozymes of malate dehydrogenase, latitudinal clines present across several continents have been identified in honey bees [9]. Natural and introduced *Drosophila* populations also exhibit similar allelic clines shown by isozyme polymorphisms of alcohol dehydrogenase (ADH) and glycerol-3-phosphate dehydrogenase (GPDH); the present study demonstrates selective pressure on these same enzymes whose expression patterns seem to correlate with latitude (reviewed by [35]). Although these markers provide an unbiased association with which to identify local adaptation, they also indicate that metabolism is often a selective target of local adaptation. Temperature influences the biosynthesis, stability and activity of proteins with functional adaptation of homologous proteins to their operating environment common [36]. While proteomics does not allow us to determine the presence of alloenzymes between populations, bi-directional segregation of pathways for metabolism and protein folding with latitude is consistent with the presence of distinct ecotypes for the warmer Californian/Hawaiian and colder Saskatchewan and Ontario populations. The Californian/Hawaiian ecotype has to deal with year round average higher temperatures resulting in the evolutionary imprinting of a lower metabolic rate and heat stress coping strategies, with opposing trends seen in Saskatchewan and Ontario populations. In order to rigorously test the hypothesis that climate and protein expression are linked, one would need to relocate honey bees adapted to specific climates to regions of similar or vastly different climates and then continue to correlate colony-level productivity with protein expression profiles.

Genomic and transcriptomic analysis are powerful tools able to dissect gene expression variations among populations (e.g. [37], [38], [39]). In *D. melanogaster* for example, 153 genes were shown to vary between natural populations sampled in Europe and Africa [40]. Gene enrichment identified genes related to the cytoskeleton being over-expressed in African populations compared to Europeans, with the opposite pattern for genes involved in fatty acid metabolism [40]. Bee transcriptome analysis has been limited to a few studies, and while none have been specifically designed to analyse inter-population variation in gene expression, relevant information can be obtained from them. For example, a recent study that focused on detecting transcript differences between healthy versus CCD bees also revealed inter-population variance [12]. By sampling bees obtained from the west and east coasts of the USA, a large amount of location specific transcript variation was detected. Gene enrichment revealed that genes controlling mitochondrial and ribosomal function were largely responsible for transcript variation, in agreement with our findings that metabolic processes are targets of local adaptation. Furthermore, a study investigating DNA methylation and gene expression status of the honey bee genome [41], found that genes encoding metabolic and energy transfer enzymes were enriched within the methylated genes. These findings reveal epigenetic imprinting potentially from environmental stimuli as a mechanism able to orchestrate changes in basal gene expression [41]. Future studies may further clarify the role of the different regulatory mechanisms responsible for the observed variations in protein levels that seem to occur in local adaptive responses of different populations.

Our findings may also open the door to expanding the use of honey bees as models of human diseases [42]. Studying honey bee populations from different origins may help us understand the differential susceptibility of human populations to metabolic diseases. Of particular interest are populations of diverse genetic backgrounds that are now living in the same environment, such as westernized populations from Eastern Europe or of Native American background. Interestingly, Fridlyand and Philipson hypothesized that the lower incidence of type 2 diabetes (T2D) in Western-Europeans, compared to westernized populations with origins in warmer climates, may be due to the existence of cold climate genes that can lead to both increased thermogenesis and decreased incidence of T2D [43]. The candidates for cold climate genes were reportedly evaluated from three areas: the uncoupling proteins, maternally-transmitted mitochondrial genes, and mitochondrial biogenesis. Given this, it is highly suggestive that in our own data mitochondrial proteins emerge as being differentially regulated in honey bee populations originated in the colder Canadian climates as compared to populations from warmer climates. These findings suggest that honey bees have similar adaptive mechanisms to humans and therefore confirm the utility of using honey bees as models of human metabolic diseases, as well as to understand the epidemiology of these diseases. In conclusion, we have provided evidence for the molecular basis of honey bee adaptations to diverse environments. Overall, energy-related mitochondrial pathways were up-regulated in bees adapted to colder climates while protein biosynthesis and degradation pathways were preferentially up-regulated in honey bees from warmer climates. The observations reported here increase our understanding of metabolic diversity in honey bee populations and lay a framework for biomarker use in selective breeding. Results may also be extrapolated to other species, confirming the need to consider the relationship of animal populations and their native biome in commercial agriculture and in natural environments. Furthermore, our findings underscore the value of honey bees as models of human diseases. Mass spectrometry- based proteomics has rarely been applied to ecology and population biology [17] but this study demonstrates that exploiting proteomics towards these goals can provide great insight into ecological issues and adaptive processes in nature.

## Materials and Methods

### Reagents

All chemicals used were of analytical grade or better and all solvents were of HPLC-grade or better; all were obtained from ThermoFisher-Scientific (St. Waltham, MA, USA). Other reagents used were purchased from the following commercial sources: Endopeptidase Lys-C, Wako Chemicals (Osaka, Japan); porcine modified trypsin, Promega (Nepean, Ontario, Canada); loose ReproSil-Pur 120 C_18_-AQ 3 µm, Dr Maisch (Ammerbuch-Entringen, Germany); 96-well full skirt PCR plates, Axygen (Union City, CA, USA); fused silica capillary tubing, Polymicro (Phoenix, AZ, USA); protease inhibitor mixture, Roche Applied Science (Basel, Switzerland); NuPAGE Novex BisTris Gels, Invitrogen (Carlsbad, CA, USA).

### Honey bee populations and sample collection

Eight populations (Table 1) of bees were used in this study and all bees were imported to and maintained at the Agriculture and Agri-Food Canada, Beaverlodge Research Farm, Beaverlodge, AB, Canada (55°18′ N; 119°17′ W) for one to two years. Multiple colonies (4–10) from each population were sampled in triplicate and five bee midguts were pooled for each sample. Midguts were dissected from the abdomens of freshly decapitated bees by using forceps to grasp the terminal abdominal segments and pulling gently. This released the almost complete digestive tract, which was then cut posterior to the crop and anterior to the rectum. Midguts were immediately washed three times to remove most of their contents and stored in phosphate-buffered saline (50 mM K2HPO4, 150 mM NaCl, pH 7.4) containing Complete, EDTA-free Protease Inhibitor cocktail (Roche) before storage at −70°C.

### Matrix for sample analysis

We generated a D-optimal design matrix [44] to group the samples in blocks of three, and assign a label to each sample (Fig 1). This randomized incomplete block design was chosen to minimize the standard error of the estimate of the population effect on protein expression level. Two constrains were included in the design: no two colonies from the same population appeared in the same block and no two samples of the same colony were assigned the same label.

### Protein preparation for mass spectrometry

With most tissues, honey bee or otherwise, we find that protease inhibitor cocktails are sufficient to prevent protein degradation. This was not the case with midgut samples, however, likely since proteolysis is one of the major functions of that tissue, and so we developed an extraction procedure where trichloroacetic acid was used to control degradation by endogenous proteases. Bee midguts were bead-homogenized in an ice-cold solution of 15% (w/v) trichloracetic acid, 1% (w/v) dithiotheitol (DTT) for 2 pulses of 2 min at 35 Hz. After 30 min on ice, precipitated proteins were collected by centrifugation at 16,100 relative centrifugal force (rcf) and the precipitate was washed 3 times with ice-cold acetone. Washed pellets were dried and solubilized in 6 M urea, 2 M thiourea, 100 mM Tris-Cl (pH 8.0); insoluble material was subsequently removed by centrifugation at 16,100 rcf. Protein estimations were carried out by a micro Bradford assay using serial dilution of BSA to establish a standard curve. Protein stability and quantity were check by 1-D Nu-PAGE (Invitrogen) and bands were visualised by staining with Coomassie Safe Blue (Pierce). For each sample, 20 µg of total protein was initially diluted to 1 µg/µl in 6 M Urea, 2 M Thiourea, 100 mM Tris-Cl, pH 8.0 and proteins were digested in solution exactly as described [45].

### Peptide clean-up and labelling

Peptide digests were purified using the C_18_ flavor of STop And Go Extraction (STAGE) tips [46] and eluted peptides were dried and labelled by reductive dimethylation using formaldehyde isotopologues [22], [47]. For each triplex, peptides were dissolved in 500 mM NaCH_3_COO (pH 8.0) and derivatized by addition of 20 µl of 200 mM CH_2_O (light) or 200 mM C^2^H_2_O (medium) and 6 µL of 1 M NaBH3CN to both or 200 mM ^13^C^2^H _2_O (heavy) and 6 µL of 1 M NaB2H3CN. The reaction proceeded for 90 min before it was quenched with 20 µL of 3 M NH_4_Cl. Samples were acidified by addition of 2% (w/v) acetonitrile, 1% (v/v) trifluoroacetic acid, 0.5% (v/v) acetic acid, then the three differentially-labeled peptide pools were combined and resolved into 5 fractions using C_18_-SCX-C_18_ STAGE tips [46]. Each fraction was dried completely and resuspended in 2% (w/v) acetonitrile, 1% (v/v) trifluoroacetic acid, 0.5% (v/v) acetic acid.

### Liquid chromatography-tandem mass spectrometry (LC-MS/MS)

Analysis of peptides by LC-MS/MS was performed using an 1100 Series nanoflow high performance liquid chromatography system (Agilent Technologies) on-line coupled to a LTQ-FT (ThermoFisher Scientific, Bremen, Germany). Peptide separation was performed by reversed phase chromatography using a 75 µm inner diameter fused silica emitter self packed with 3 µm Reprosil-Pur C_18_-AQ resin (Dr. Maisch GmbH). Peptides were loaded in 4.8% (v/v) aceonitirle, 0.5% (v/v), acetic acid at 0.6 µL/min and then resolved at 200 nL/min for 75 min, during which a linear gradient of acetonitrile was created from 4.8% to 64% in 0.5% (v/v) acetic acid. Mass spectrometry: Operating in data dependent aquisition, the LTQ-FT was set up to aquire FT full scan data over a mass range of 350–1600 m/z before performing FT selected ion monitoring (SIM) and MS/MS in the ion trap on the top 3 most intense multiply charged ions [48].

### Protein identification and quantification

Peak lists were created using DTASuperCharge [49] with default parameters and searched using Mascot (v2.2) against the Honey Bee, *A. mellifera* Amel_4.0 translation (forward plus inverted sequences) of the genome with additional entries for human keratins, porcine trypsin and LysC. Tryptic cleavage rules (R/K, except preceding P) were specified with up to two missed cleavages allowed. Carbamidomethyl (C) was set as a fixed modification, Acetyl (Protein N-term), Deamidated (NQ), Oxidation (M), Dimethyl (K), Dimethyl (N-term), Dimethyl:2H(4) (K), Dimethyl:2H(4) (N-term), Dimethyl:2H(6)13C(2) (K), Dimethyl:2H(6)13C(2) (N-term) as variable modifications. Peptide tolerance was set to 10 ppm and MS/MS tolerance was 0.6 Da. The false discovery rate (FDR, % of type one errors) for peptide identifications was estimated at 0.25% for the whole dataset as: FDR = rev/(for+rev) where ‘rev’ are the number of hits against reversed protein sequences and ‘for’ are the number of hits against real protein sequences for a given cut-off criteria. All peptides with an IonsScore ≥25 were quantified using MS Quant (v1.5) [49]; after automated quantitation all files were manually edited to ensure consistent quantitation and the peak area ratios were exported for further analysis. An in-house script, finalList.pl, described previously [47] for applying parsimony (Occam's razor) to generate a non-redundant list of identified proteins from a large pool of independent experiments was adapted to simultaneously calculate average peptide ratios for each protein in each block.

### Statistical analysis

Logarithms of intensities were normalized by first subtracting the average of the three measurements in each block (for each protein independently) and then centering and standardizing within each label (across proteins) by the median and median absolute deviation. For each protein, a Linear Mixed Effects model was used to estimate the effect of population on the protein expression level, adjusting for block and label factors. Colony was treated as a random factor to control for the three repeated measures within each colony. Proteins for which the population factor was significant at *P*<.05 were selected for further analysis. For the significant proteins the following analysis was performed: for each protein, individual effects of the 8 populations and their standard errors were computed (keeping the average effect equal to 0). They were then converted into a set of 8 one-sided *P*-values such that values close to 1 indicate strong positive effect and values close to 0 indicate strong negative effect. *P*-values were chosen over z-values so that all strongly expressing populations were grouped together regardless of the degree of expression. One-sided *P*-values were chosen over 2-sided so that the directionality of the change in expression was carried forward. To convert 1-sided *P*-values into the more traditional 2-sided *P*-values the following formula can be used: *P*2 = 1−2*|*P*1−0.5|, which can be visualized as an inverted V-shape centered around 0.5. All calculations were performed in R.

### Gene ontology enrichment and expression overlap and clustering

Gene ontology (GO) enrichment analysis was performed based on the *Drosophila* orthologs to the complete protein sequence of the bee proteins identified. GOToolBox [50] was used to calculate enrichments between protein lists of interest using the entire midgut proteome characterized here (578 proteins) as background. A hypergeometric test with subsequent correction for false discovery rate (FDR) when using multiple testing was applied. To determine statistical significance of protein overlaps between populations a representation factor was calculated: (number of significant proteins common between two populations)(number of proteins identified)/(number of proteins in population X)(number of proteins in population Y) [51]. Overlap for all binary relationships possible was calculated and statistical significance tested using exact hypergeometric test (1-tailed) and where *P*<.05 GO categories for overlapping proteins were determined as done previously. SOTA (self-organizing tree algorithm) clustering was used to determine one side probability metrics for all 172 population significant proteins across all eight honey bee populations. Using MultiExperiment Viewer [52] eight hard clusters were generated using seven cycles with a maximum diversity of 0.8. Hierarchical dendrograms for population and proteins were calculated using Euclidean distances.

## Supporting Information

Dataset S1 Spreadsheet containing the randomized incomplete block design (RIBD). Columns show how each colony (4–6) from different populations (1–3) is distributed across 58 blocks using a triplex experimental design afforded by the use of the triplex dimethylation labeling strategy.
(0.02 MB XLS)

Dataset S2 Spreadsheet containing the raw quantitative ratios observed for each protein (columns) identified in the 58 blocks (three colonies per block) analyzed. The top row indicates the bee colonies in each block.
(1.94 MB XLS)

Dataset S3 Expression spreadsheet of quantified midgut proteins, consisting of column A: the Honey bee gene identifier accession number from the NCBI Genbank database, column B: the *Drosophila* refseq accession number from the fly NCBI reference sequence database, column C: the Flybase accession number, column D: the K code from the Kyoto Encyclopedia of Genes and Genomes database, columns E-L: the 1-sided P values and columns M-T: stock effect values for each population and protein analyzed. The 2-sided P value for population effect for each protein is given in column U and V states if this value was considered significant. Columns W-AC give up to three example peptide sequences with MASCOT scores that were quantitated for each protein identified.
(0.36 MB XLS)

Dataset S4 Complete gene enrichment spreadsheet for all analysis performed and shown in the results section. Each sheet has self explanatory column headers, sheet 1 ‘Table 1’ is the full results of the data summarized in Table 1. Sheet 2 ‘Figure 3’ is the enrichment results based on the similarity matrix present in Figure 3. Sheet 3 ‘Figure 4’ is the enrichment results based on the top 4 clusters presented in Figure 4.
(0.05 MB XLS)
